# The rising prevalence of obesity: part B—public health policy solutions

**DOI:** 10.1097/IJ9.0000000000000019

**Published:** 2017-06-22

**Authors:** Maliha Agha, Riaz Agha

**Affiliations:** a Department of Primary Care and Public Health King’s College London;; b Department of Plastic Surgery, Guy’s and St. Thomas’ NHS Foundation Trust, London, UK

**Keywords:** Obesity, Overweight, Public health

## Abstract

Obesity is likely to supersede tobacco as the biggest cause of premature death. England has some of the worst figures and trends in obesity compared with the rest of the Europe. Rising obesity prevalence is an international crisis that has the potential to overwhelm health care resources as well as creating enormous human suffering and social cost. This article outlines potential public health policy solutions to this crisis.

## Policies to prevent obesity

An effective public health policy to reduce obesity should be based on the following principles^1^:

- Reliable predictions of the future.
- Understanding what policies will be feasible on a long-term basis.
- Consequences related to the policy (particularly environmental).

Determining the root cause of obesity is central to any effort to prevent it^2^. Designing effective strategies to prevent and treat obesity is difficult due to the uncertainty around the etiology of obesity^3^.

Environmental conditions serve as a “default” for affecting the behavior and health of the population. For instance poverty and social inequality can be among the root cause for poor access to health care, inadequate education, poor living conditions, and stress, therefore affecting health. Greater exposure to such “defaults” produces increased health consequences^4^. Policies to change defaults are, therefore, more effective and cheaper than the traditional means of persuasion and education. For example, banning smoking in public places in 2008 resulted in a significant reduction in smoking^5^.

Policies to reduce obesity should focus on promoting children’s health, nutritious food, physical activity, supporting health in the workplace, and behavioral modification (self-monitoring of eating habits, stress management, stimulus control, problem solving, contingency management, cognitive restructuring, and social support). Policies to provide effective treatment and support to overweight and obese people must also be implemented to reduce health inequalities too^6^.

Health targets must be set for each policy between the associated bodies to monitor the effectiveness of the policy and to ensure concentrated efforts and uniformity of policy implementation (but rules must be implemented to achieve the target by fair means and without bias).

## Schools as an important starting point

Youth obesity is due to environmental defaults such as lack of activity, poor nutrition, and the ready availability of large portions of calorie-dense food. Schools often contribute to the creation of a negative food and activity environment, thus setting poor defaults for children’s health and nutritional understanding and awareness. In the United Kingdom, 98% of secondary schools sold soda, 78% sold cookies, and 69% sold potato chips, in 2006^7^–^11^.

Evidence shows that school-based policies can be effective in improving the health of school children. However, policies must be carefully constructed due to the prevalence of weight-bias in schools^12^. Prohibiting junk food and banning vending machines in schools and soft drink advertisements in the cafeteria and promoting school-based lunch programs (such as those popularized by the Chef Jamie Oliver) has shown to lower students body mass index (BMI)^13^–^15^. Actively promoting young children to consume fruits and vegetables during meals (rather than leaving them to help themselves) has been shown to significantly increase their consumption^16^.

## Nutritional environment

The United States has a significantly greater rate of obesity when compared with Japan—what lessons can be learnt from this? The food culture in Japan has sufficiently resisted some of the global trends in obesity^17^. The cheapness, easy availability, highly palatability, increased fat-consumption, commercial food promotions, reliability on snacks and ready-made meals, and heavy marketing of energy-dense foods results in increased food intake and decreased consumption of healthy foods. However, the food industry has always blamed lack of physical activity among obese individuals as the main contributor to obesity^18^. In the 1940s each kilojoule of carbohydrate in the diet had 0.6 kJ of fat, increasing to 0.9 kJ in the 1990s which is an increase of 50%.

Increasing the accessibility of a diet rich in fresh vegetables, fruits, and lean meats must be made to replace processed and fast-food rich diets. Buy-one-get-one-free schemes must be used more on nutritious than non-nutritious foods. Food labelling must be improved by providing the correct nutritional properties of the food in an easy to read format. Labels that claim “low in salt” or “light” will have to meet the standardized definitions agreed by the EU. Food high in fat or sugar must be labelled as such. The use of “traffic light labels” (high, medium, or low amounts of fat, saturated fat, sugars and salt per 100 g of the food) must be corrected for those manufacturers using it in the form of a percentage showing an individual’s Guideline Daily Amount of a nutrient which is wrong.

For any broad nutritional advancement, wholesale cultural and societal changes are required. Food industries need to take responsibility for their products to place genuine efforts to reform the nation’s diet^19^. Policies to target behavior by reducing the sugar in children’s beverages can also show promising results^20^. Therefore, by changing the supply chain and influencing those who govern and monitor changes in health, the targets can be achieved significantly.

Evidence suggests that a growing number of British people lack the basic skills to prepare a healthy meal. Therefore, by giving people a better understanding of healthy eating through training we can help change the dietary milieu of the population.

## Physical activity

According to the Royal College of General Practitioners, on an average food intake has fallen by 750 kcal/d and activity levels decreased by 800 kcal/d, thus contributing to rising obesity. The tackling obesity in England report reveals that, compared with today, the extra physical activity in daily living 50 years ago was equal to running a marathon a week. An average person now walks 189 miles/y compared with 255 miles over 25 years ago. We are walking less than we have ever done in history. This is due to replacement of active modes of transport (walking and cycling) with motorcars, buses, tubes, trams, and trains, etc. Physical activity in adults delays cognitive impairment in the elderly^21^,^22^ and also reduces cardiovascular risk factors, diabetes, and some cancers^23^.

Three reviews have concluded that decrease in total energy expenditure is the main reason behind the current obesity epidemic^24^–^26^. Although there has been an increase in self-reported leisure time physical activity, evidence shows a decrease in physical activity in nonleisure time, such as occupation, transportation, and household chores^27^. Therefore, to promote and encourage an active lifestyle through the provision of safe sidewalks or safe cycle lanes a redesign of urban infrastructure is required.

At work, employers can support their employees by providing healthy meal options in cafeterias, making shower facilities available for those cycling to work and providing gym memberships to employees.

## Education

Evidence shows a decrease in professional advice on losing weight among all ages with the most noticeable decline among older adults^28^. Although healthy eating recommendations have been developed for many years, people are still not making healthy choices on how much and what to eat. According to the Food Standards Agency, there is still a high intake of saturated fat, sugar, and salt and lower than recommended intake of fruits and vegetables. This shows that health promotion messages are not delivered loud or consistently enough to change the behavior of people. Frequent professional advice and counselling to overweight and obese people will play a significant role in increasing awareness of the risks associated with obesity. Drug therapy and surgery in extreme cases can also make a significant contribution in tackling obesity.

## Advertisements

Education and lessons work as a catalyst to help change behavior. However, their decision-making is heavily counterbalanced by advertising and promotion campaigns undertaken by the food industry^29^. In United Kingdom, children aged 15 to 18, watch over 2.5 hours of television per day^30^. Marketing a brand or a product has a great psychological and emotional power on the population. A Mintel study on advertising costs revealed that only a fraction of money is spent on advertising fruits compared with chocolates, snacks, etc. Also the £5 million annual budget of the Government’s Five-a-day campaign is simply drowned out by the advertising budgets of large food companies with their own agenda (**Tables 1**, **2**)^31^.

**Table 1:**
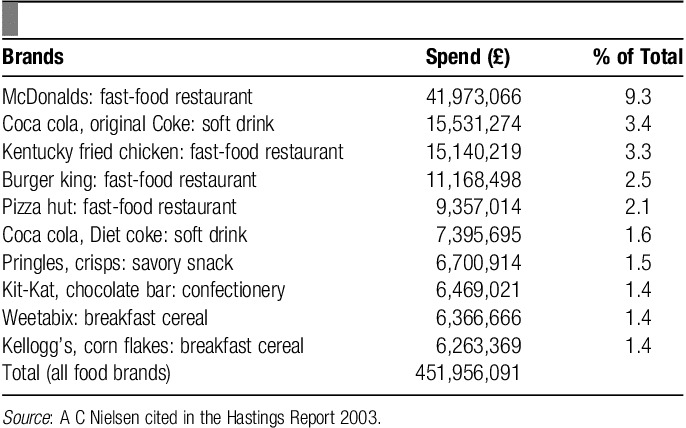
Advertising spend across the top 10 advertised food brands in the United Kingdom (2002).

**Table 2:**
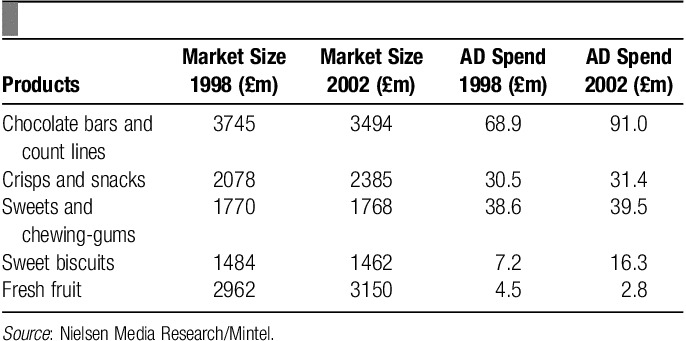
Children’s snack products, market size, and advertising (AD) spend (1998–2003).

It was also found that food advertisements were shown more frequently during children’s programs (45%–58%) compared with adult programs in the United Kingdom (21%). Of these advertisements, 95%–99% of food advertisements were high in fat, sugar, or salt and 86% during adult programs. However, no adverts for fresh fruits and vegetables were shown during either of the programs^16^. This shows that children are targeted more intensively when promoting and advertising ultimately unhealthy foods. One report showed that for soft drinks companies, 8-year-old boys are the ideal target customers^16^.

Like Sweden, Norway, and Quebec government policy, advertisements aimed at children under 12 or during children programs should not be permitted in other countries. Like in Finland, Denmark, and Netherland, presenters from children’s programs must not appear in advertisements. Fast foods such as McDonald’s should be restricted from promoting toys in its advertisements in the United Kingdom^32^. The same advertisement strategy can be used to replace attitude by replacing non-nutritious food advertisements with advertisements focusing on promoting and increasing knowledge about fruits and vegetables.

## Fruit and vegetable prices

Fruits and vegetables are considerably cheaper in street markets than in supermarkets. Therefore, trimming the profits attached to fresh fruits and vegetables in the supermarkets can also bring some considerable scope^33^.

## BMI monitoring

BMI screening is essential in every age group and can lead to changes in behavior. A screening program in London showed overweight girls restricted their unhealthy diet and around 50% showed a positive change in their behavior. Recent studies indicated that school-based BMI screening is generally accepted by British^34^ and American^35^ children and their parents. This may be because parents often underestimate their children’s weight and would like to be informed of their weight status. However, a small percentage of adverse effects such as embarrassment and shame were reported by the children.

Policies for frequent BMI screening (in schools particularly) must be implemented^36^, along with control over the consequences associated with it such as weight-related teasing, stigmatization, eating disorder risk, parental efficacy and educational quality^37^.

## Government and health sector

**Figure 1:**
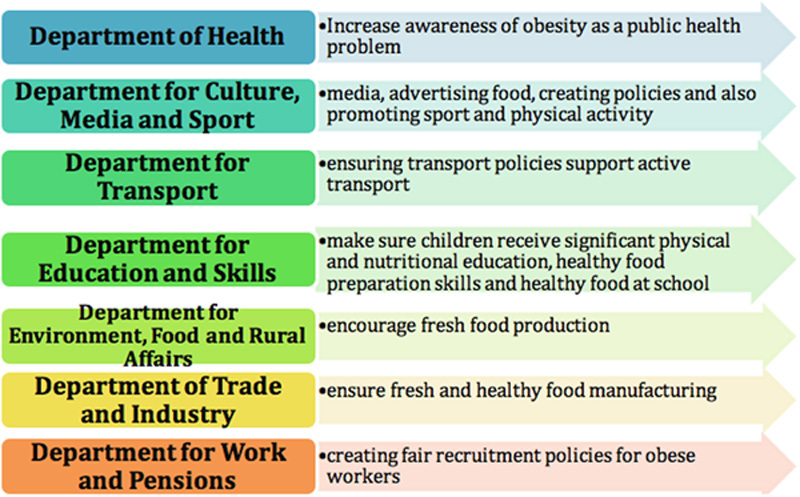
Government departments.

Changing the economic, social, and political environment which “fosters” obesity will not be easy. It will require determination and resolve from government leaders to create and maintain change. Government should concentrate its efforts on informing the population and addressing environmental factors to make healthy choices easier^38^. The associated government departments need to work together to impact obesity (**Fig.** **1**)^39^.

The older population are usually considered the most reticent to change their long-standing health behaviors. Therefore, health promotion programs for the older population should be implemented and nutritional policies for older adults must focus on providing meals and social educational services to older people^40^. We have talked about interventions for preconceptual and pregnant women elsewhere^41^.

## Conclusions

Public health policies are significantly required to address the behavioral, environmental, and sociocultural factors creating the “obesogenic” environment by promoting decreased calorie intake and increased physical activity.

However, due to the continuous increase in the prevalence of obesity and lack of evidence for efficacy, it is difficult to measure which policy interventions will be most effective at halting the rise of obesity.
